# Concomitant analyses of intratumoral microbiota and genomic features reveal distinct racial differences in breast cancer

**DOI:** 10.1038/s41523-023-00505-6

**Published:** 2023-01-26

**Authors:** Sheetal Parida, Sumit Siddharth, Yuqing Xia, Dipali Sharma

**Affiliations:** Department of Oncology, Johns Hopkins University School of Medicine and Sidney Kimmel Comprehensive Cancer Center at Johns Hopkins, Baltimore, MD USA

**Keywords:** Breast cancer, Metagenomics

## Abstract

Racial disparities are most accentuated among Black women as their lifetime risk of breast cancer incidence is lower than white and Asian women but their breast cancer related mortality is the highest among all races. Black women are more likely to develop triple-negative breast cancer at a younger age and harbor more aggressive tumors. In addition to tumor-centric alterations, tumor growth is also influenced by multiple other tumor microenvironment-related features, including resident immune cells and microbiota. Hence, in this study, we conduct concurrent genomic and metagenomic analyses, and uncover distinctive intratumoral microbial community compositions and tumor immune microenvironment-related traits in breast tumors from Asian, Black and white women. Interestingly, unique racially associated genomic nodes are found in the breast tumors from Asian, Black and white women. Examination of the cellular heterogeneity show differential enrichment of 11 out of 64 immune and stroma cell types in the breast tumors from different racial groups. In terms of microbial diversity, significant differences are revealed in alpha and beta-diversity measures. Intriguingly, potential race-specific microbial biomarkers of breast cancer are identified which significantly correlate with genes involved with tumor aggressiveness, angiogenesis, tumor cell migration and metastasis as well as oncogenic pathways-GLI and Notch. Investigating the metabolic features of intratumoral microbes, we find a significant differential enrichment of environmental information processing pathways, oncogenic pathways, and lipid metabolism pathways. Concomitantly investigating tumor-centric, tumor immune microenvironment-related and microbial alterations, our study provides a comprehensive understanding of racial disparities in breast cancer and warrants further exploration.

## Introduction

Approximately 287,850 new cases of invasive breast cancer and 51,400 new cases of in situ breast cancer will be diagnosed in US women in 2022 as the risk for developing invasive breast cancer has increased to about 1 in 8 US women in her lifetime. It is worth noting that breast cancer risk and outcomes differ greatly across races and ethnicities. White women have the highest lifetime risk of breast cancer incidence (13%) in comparison to American Indian & Alaska Native (8%), Asian & Pacific Islander (11%), Black (12%), and Hispanic (11%) women^1^. Of note, the risk of breast cancer incidence in younger Black women (under the age of 45 years) is much elevated compared to their age-matched white counterparts. Despite significant reduction in overall breast cancer related mortality, Black women are at a 40% higher risk of succumbing to breast cancer^2^. Historically, incidence rates of breast cancer in Asian women have been lower compared to their western counterparts, however, in the past four decades, breast cancer incidence has exponentially increased in Asia and Southeast Asia^3^. Interestingly, immigrant Asian American women are 2.46 to 3 times more likely to encounter breast cancer compared to US born Asian American women^4^. Asian women develop breast cancer at a younger age (40–49 years) than white women, whose probability of developing breast cancer peaks at 70 years of age^3^. Black women in US also develop breast cancer at a younger age (40–45 years) but they are highly likely to develop triple-negative breast cancer (TNBC) whereas younger Asian women are more prone to developing Luminal B subtype with TP53 mutation^3,5^. Various biological and socioeconomic factors contribute to racial disparities in breast cancer incidence and outcomes^6,7^. Indeed, socioeconomic status is one of the major determinants of insurance coverage, access to primary care, timely referrals, health and nutrition, comorbidities, post-treatment follow-up, mental health issues and preventative measures like mammograms, and these variables directly or indirectly contribute to racial disparities^8–12^. Somewhat contrasting with the impact of the aforesaid socioeconomic drivers, studies have shown that even under equal access scenarios, disparities in breast cancer incidence and outcomes are evident^13,14^ especially for hormone negative or TNBC^15–19^. Multiple clinical and translational studies have highlighted the underlying biological differences in breast tumors among different races^20–23^, but the biological underpinnings of racial disparities are still elusive.

The factors governing racial disparities in breast cancer are indeed multifactorial and it is pertinent to identify additional modifiers of racial disparities. Recently, the microbiota has gained prominence as an important regulator of the tumor incidence and progression as well as tumor microenvironment and immune landscape. Breast tumors are known to harbor intratumoral as well as intracellular microbes. In fact, breast tumors and associated immune cells harbor the most biodiverse and rich microbiota among many other cancer types examined^24^. The microbiota potentially determines the fate of the tumor by modulating systemic inflammation, regulating local immune responses, synthesis of signaling peptides and genotoxins^25–30^, inducing DNA damage thereby driving genomic instability^31^, xenobiotic metabolism and drug detoxification^32,33^, as well as regulation of the steroid hormone levels in the body^34–36^. Recent studies have also shown that the microbiota is more heritable than once thought^37–39^. Although a limited number of studies have provided evidence to support the alterations in microbial community composition in normal breast and tumors, and two recent studies presented racial differences in breast microbiota^40–44^, a major drawback of the microbiota-related studies has been the small sample sizes.

The interaction between the metagenome and the host genome is the subject of intense investigation and could be instrumental in greatly improving the mechanistic understanding of variable outcomes among different races. Importantly, the microbiome is cumulatively shaped by the diet, lifestyle, geography, exposure to antibiotics, drugs or toxins, chronic diseases as well as genetics; and many of these factors are also associated with socioeconomic status and race. Hence, we aim to provide an insight into the distinctive microbial communities in the breast tumors from Asian, Black and white women and how it might be shaping the tumor microenvironment. To mitigate the issue of small sample sizes inherently associated with microbiota-related studies, we examine the genomic and metagenomic data from The Cancer Genome Atlas (TCGA) cohort encompassing 1018 breast cancer patients categorized into Asian, Black and white groups (self-reported race in accordance with the U.S. Census Bureau and National Institute of Health). Our study uncovers unique genomic nodes, differential enrichment of immune and stroma cell types, and potential race-specific microbial biomarkers in different racial groups. Furthermore, race-specific microbial biomarkers are associated with distinct genetic pathways and metabolic features.

## Results

### Breast tumor microenvironment possesses distinct cellular and supracellular patterns among different races

**Fig. 1:**
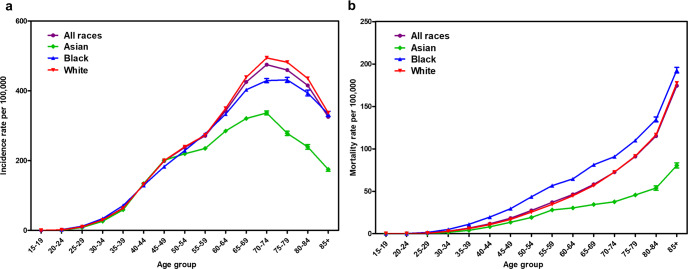
Mortality associated with breast cancer is highest in Black women. **a**, **b** Age-specific incidence and mortality rates of all breast cancers in US women from 2000 to 2017 as per SEER database.

With an overall goal to examine the biological underpinnings of racial disparities in breast cancer, we started this study by investigating the SEER (The Surveillance, Epidemiology, and End Results Program) data to underscore the racial differences in breast cancer incidence and survival among different races. The incidence of breast cancer is the highest among white women (131.8 per 100,000) followed by Black women (124.7 per 100,000) and Asian women (105.1 per 100,000) (SEER 21, 2014–2018, Age adjusted) (Fig. 1a). However, the death rates are the highest among Black women (27.1 per 100,000) followed by white women (19.4 per 100,000) and Asian women (11.6 per 100,000) (SEER U.S.2015–2019, Age-Adjusted) (Fig. 1b).

**Fig. 2:**
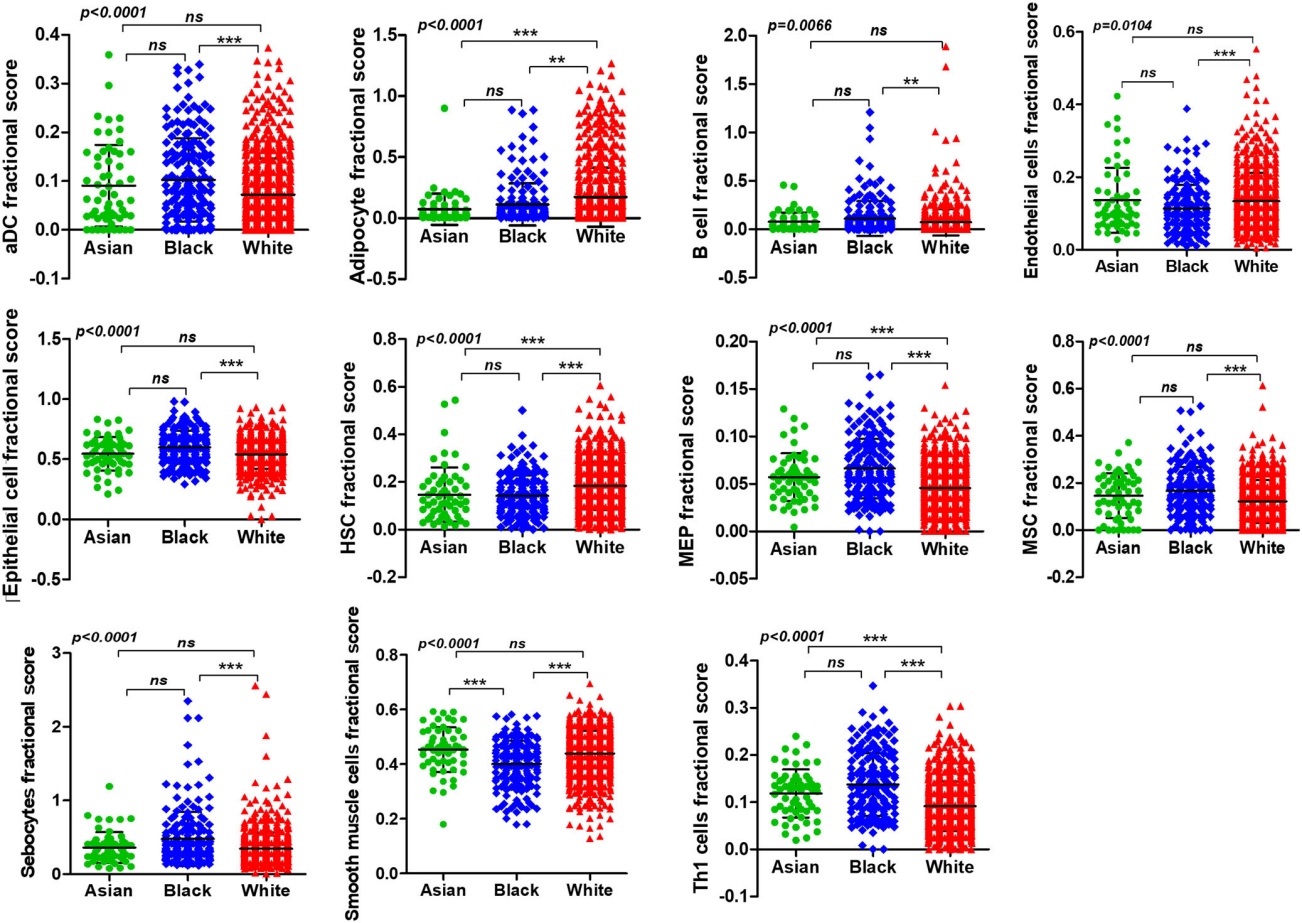
Breast tumors are heterogenous and are composed of multiple cell types. xCell analysis of the TCGA data for all breast cancer. Cell types showing significant differences between breast tumors from women of different races are shown. Differences estimated by Kruskal–Wallis test followed by Dunn’s post-test between all pairs of samples. Indicated *p*-value represents *p*-value summary of one-way ANOVA and asterisk represent *p*-values between indicated groups. *p* < 0.0001***, *p* < 0.001**, *p* < 0.05* (*χ* ^2^ test). Error bars represent standard deviation (SD).

Breast tumors are heterogeneously composed of the stroma, immune cells, blood vessels and the extracellular matrix. To get a comprehensive picture of the dissimilarities in the tumor microenvironment composition between different races, we used the TCGA data for all breast cancers and analyzed their 64-cell type signature using web-based tool, xCell. The comparative cellular landscape of breast tumors from Asian, Black and white women is presented as a heat map in Supplementary Fig. 1. Intriguingly, 11 out of the 64-cell types showed very distinct and statistically significant variation among races (Fig. 2) (By one-way ANOVA). The cellular differences were most pronounced between tumors from Black and white women, while the tumors from Asian and Black women showed the least definitive differences compared to each other. The tumors from Black women had significantly higher levels of activated Dendritic Cells (aDC), B cells, epithelial cells, Megakaryocyte–erythroid progenitors (MEP), Mesenchymal Stem Cells (MSCs), Sebocytes and Th1 cells, and significantly lower proportions of Endothelial cells, Hematopoietic Stem cells (HSC) and Smooth muscle cells compared to the tumors from white women. The only significant difference between the tumors from Asian and Black women was the higher proportion of Smooth muscle cells in the Asian group in comparison to the Black group. Another important component of the tumor microenvironment, adipocytes, showed least accumulation in the tumors from Asian women while their proportion was significantly higher in tumors from white women in comparison to Asian and Black women. Among the Asian and white group, proportion of HSCs was significantly lower in Asian group while MEPs and Th1 cells were significantly higher in tumors from Asian women (Fig. 2). Th1 response is absolutely important in the context of tumor immunology and immunotherapy. IFNγ produced by stimulated Th1 cells is known to direct macrophages to induce cytotoxicity^45^. While Th1-associated cytokines IL1β, IL6, IL2, and IL12 did not show significant difference, IFNγ was indeed significantly higher in tumors from Asian women compared to tumors from white women (Supplementary Fig. 2). Th1 cells induce macrophages to produce cytotoxic CXCL9 and CXCL10 within tumors. While CXCL10 varied insignificantly across races, CXCL9 was significantly overexpressed in tumors from Black women compared to tumors from white women (Supplementary Fig. 2). These results reveal that breast tumors from Asian, Black and white women have certain specific cellular patterns in the tumor immune microenvironment.

### Metagenomic analysis uncovers distinctive microbial community composition in breast tumors from Asian, Black, and white women

**Fig. 3:**
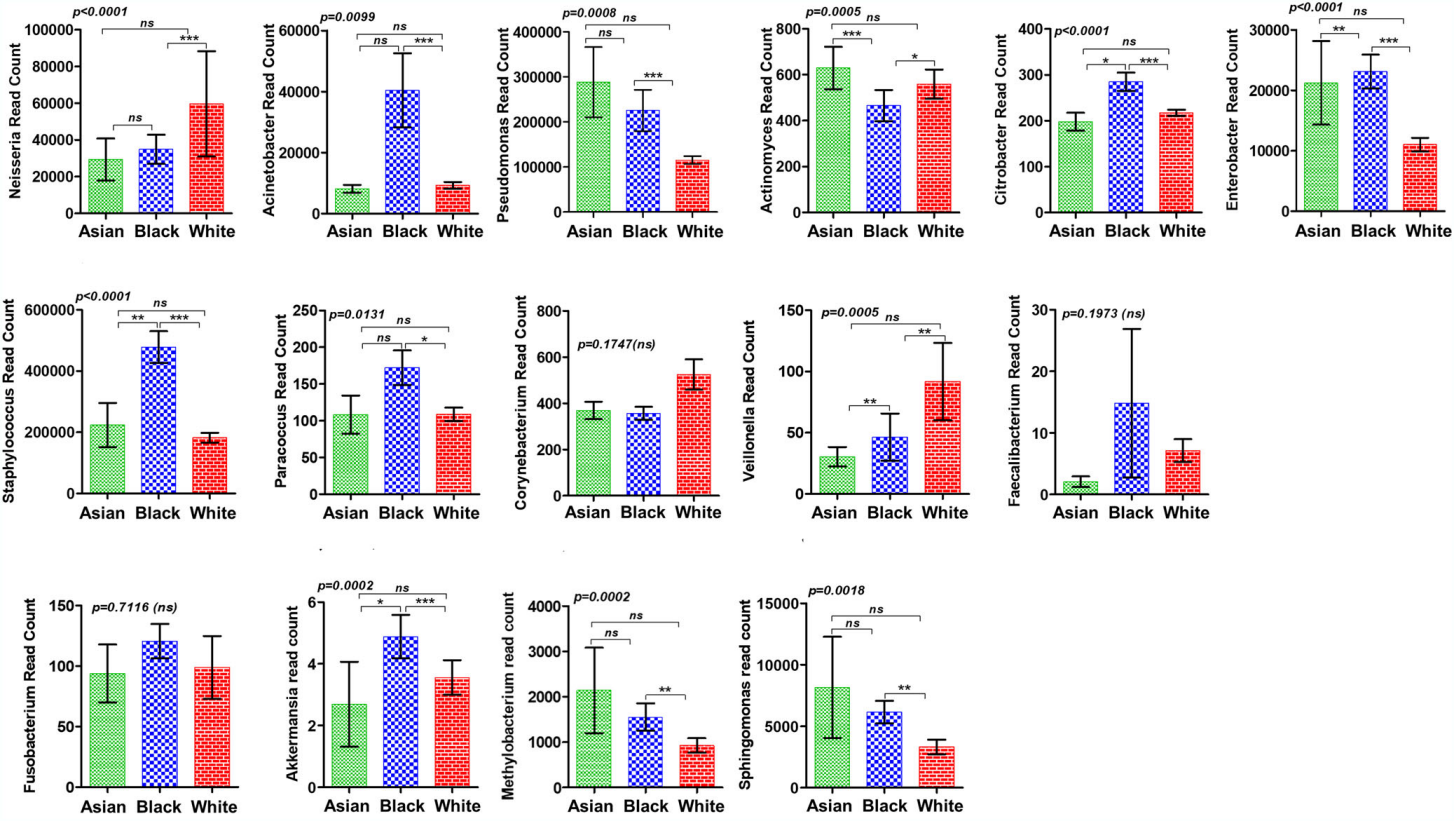
Breast tumors from different races harbor differential levels of tumor-specific bacteria. Bar graphs show the differences in the levels of selected bacterial genera known to be associated with breast tumors in different races. Differences estimated by Kruskal–Wallis test followed by Dunn’s post-test between all pairs of samples. Indicated *p*-value represents *p*-value summary of one-way ANOVA and asterisk represent *p*-values between indicated groups. *p* < 0.0001***, *p* < 0.001**, *p* < 0.05 *(*χ* ^2^ test). Error bars represent standard deviation (SD).

The microbiota has emerged as a major component of the tumor microenvironment. A recent seminal study showed that breast tumors are the richest in terms of bacterial richness and diversity^24^. In addition to residing within tumor cells, microbes also harbor inside CD45+ immune cells indicating their plausible influence on the intratumoral immune modulation. Two recent studies have examined the bacterial community composition in tumors from non-Hispanic Black and non-Hispanic white women revealing some interesting patterns but were limited by a small sample size^40,41^. We aimed to investigate the differential microbial composition of breast tumors from different races but prior to the comprehensive analysis of microbiota, we examined the levels of bacterial genera proven to be over-represented in breast tumors compared to normal breast tissue in breast tumors from different races (Fig. 3). Estimated by Kruskal–Wallis test followed by Dunn’s post-test between all groups, most of bacterial genera queried indeed varied significantly between races, especially Black and white (Fig. 3) groups.

**Fig. 4:**
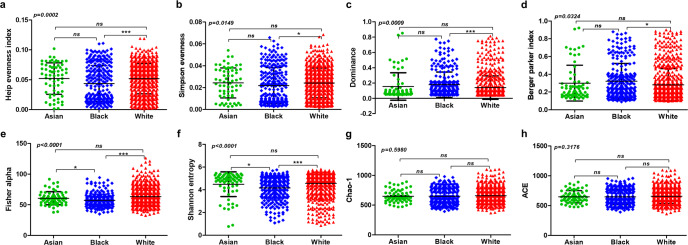
Alpha-diversity measures: significant difference in community evenness is evident between breast tumors from different races. Comparison of the bacterial community composition of 1018 primary tumors encompassing tumors from Asian (*n* = 65), Black (*n* = 257), and white (*n* = 696) women. **a** Heip evenness, **b** Simpson evenness, **c** Dominance, **d** Berger-parker index, **e** Fisher alpha, **f** Shannon entropy, **g** Chao-1, **h** ACE matrices. Differences estimated by Kruskal–Wallis test followed by Dunn’s post-test between all pairs of samples. Indicated *p*-value represents *p*-value summary of one-way ANOVA and asterisk represent *p*-values between indicated groups. *p* < 0.0001***, *p* < 0.001**, *p* < 0.05 *(*χ* ^2^ test). Error bars represent standard deviation (SD).

**Fig. 5:**
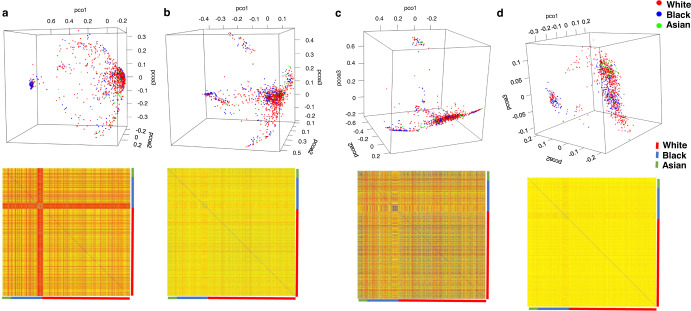
PCoA plots and corresponding heatmaps show beta-diversity between tumors from different races. Comparison of the bacterial community composition of 1018 primary tumors encompassing tumors from Asian (*n* = 65), Black (*n* = 257) and white (*n* = 696) women. **a** Bray–curtis, **b** Jensen-Shannon, **c** Jaccard, and **d** correlation matrices.

Encouraged by these observations, next, we analyzed and compared the bacterial community composition of 1018 primary tumors encompassing tumors from Asian (*n* = 65), Black (*n* = 257), and white (*n* = 696) women. Alpha-diversity measures highlighted that the breast tumor microbiota in Asian women did not significantly vary from either Black or white women. Notably, Heip evenness (*p* = 0.0002) (one-way ANOVA), Simpson evenness (*p* = 0.0149), Dominance index (*p* = 0.0009), and Berger-parker index (*p* = 0.0324) (one-way ANOVA) were significantly different between breast tumors from Black and white women. Chao1 index and ACE did not show any significant differences but Fisher alpha and Shannon entropy showed that the microbiota composition between breast tumors from Black and white women were significantly diverse (*p* < 0.0001) (one-way ANOVA) (Fig. 4). Next, we investigated the beta-diversity among breast tumors from Asian, Black and white women. Figure 5 shows the principal coordinate analysis (PCoA) plots and heatmaps representing the beta-diversity analysis between breast tumors from the women of three races based on Bray–Curtis, Jensen-Shannon, Jaccard, and correlation matrix. While the data points belonging to the tumors from Asian women remained dispersed throughout, the data points representing tumors from Black and white women segregated into two distinct clusters suggesting that there might be significant differences in bacterial community composition of breast tumors from Black and white women (Fig. 5).

**Fig. 6:**
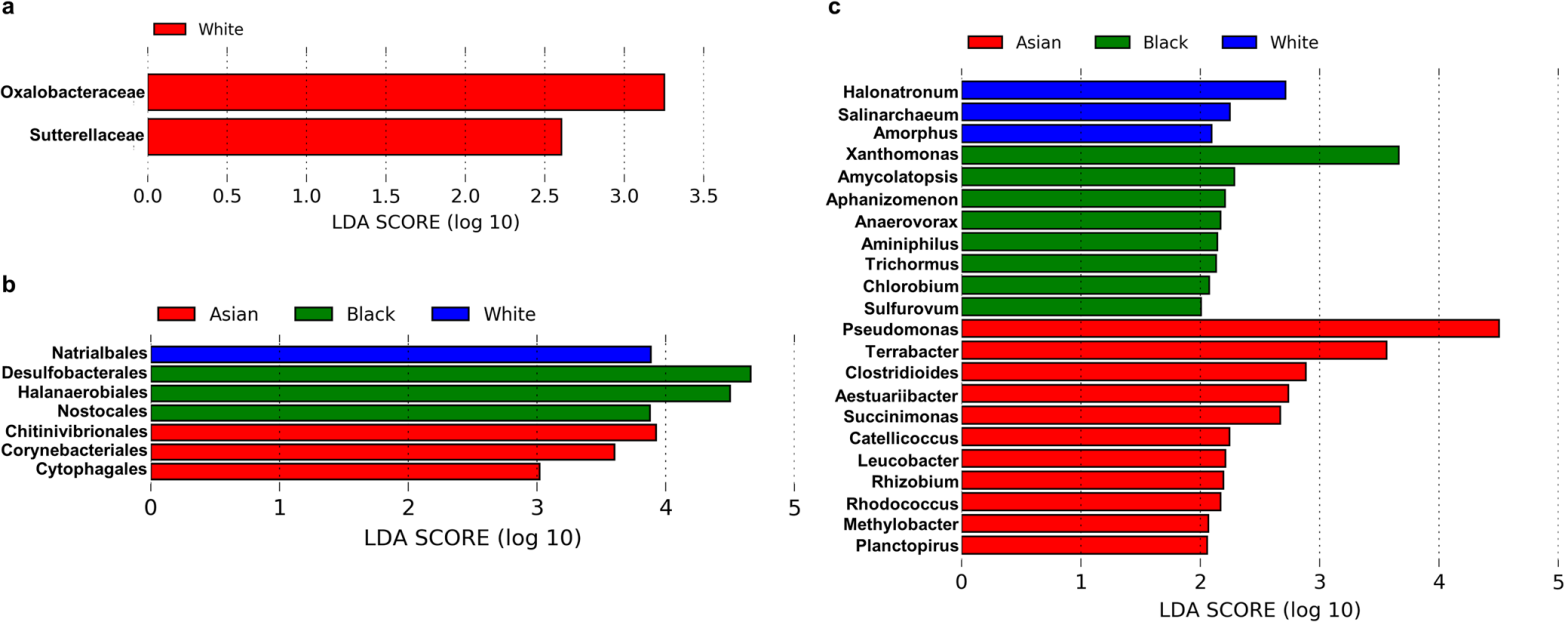
Several microbial biomarkers are associated with breast tumors from Asian, Black, and white women. Linear discriminate analysis (LDA) scores are utilized to predict microbial biomarkers in 1018 primary tumors encompassing tumors from different races. Plots show **a** Order, **b** Family, and **c** Genus.

To identify the potential microbial biomarkers among different races, we performed the Linear discriminant analysis Effect Size (LEfSe) (Fig. 6). At the level of order, only two orders, Oxalobacteraceae and Sutterellaceae could be distinguished in breast tumors of white women (Fig. 6a). Using an LDA cutoff score of 2, at family level, Natrialbales was identified in tumors from white women; Desulfobacterales, Halanaerobiales and Nostocales in tumors from Black women; and Chitinivibrionales, Corynebacteriales and Cytophagales in tumors from Asian women as potential biomarkers (Fig. 6b). At the genus level, Halonatronum, Salinarchaeum and Amorphus were identified in breast tumors from white women; Xanthomonas, Amycolatopsis, Aphanizomenon, Anaerovorax, Aminiphilus, Trichormus, Chlorobium, Sulfurovum were noted in breast tumors from Black women; and Pseudomonas, Terrabacter, Clostridiodes, Aestuariibacter, Succinimonas, Catellicoccus, Leucobacter, Rhizobium, Rhodococcus, Methylobacter and Planctopirus emerged as potential biomarkers in breast tumors from Asian women (Fig. 6c).

**Fig. 7:**
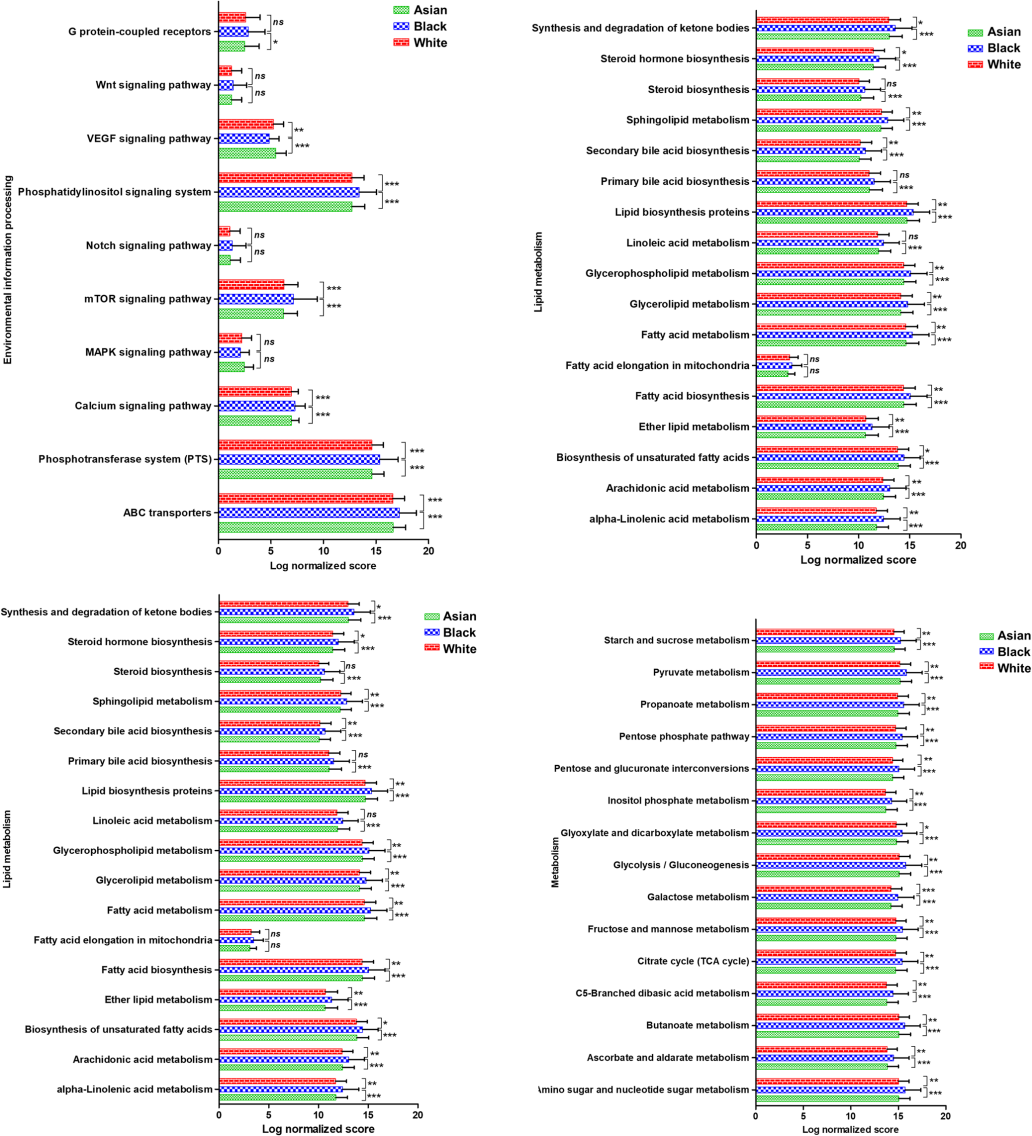
Selected KEGG pathways are enriched in breast tumors from different races. A total of 1018 primary tumors encompassing tumors from different races were analyzed using PICRUSt analysis. Plots show the enrichment of various pathways. Indicated *p*-value represents *p*-value summary of two-way ANOVA and asterisk represent *p*-values between indicated groups (*χ* ^2^ test). All comparisons were non-significant for Asians vs. white groups. Error bars represent standard deviation (SD).

Next, we evaluated the probable metabolic contributions of the tumor-specific microbial communities utilizing Phylogenetic Investigation of Communities by Reconstruction of Unobserved States (PICRUSt) analysis. PICRUSt analysis showed an enrichment of 309 KEGG pathways in microbiota of breast cancer across the three races including pathways and processes related to cellular processes, environmental information processing, genetic information processing, human diseases, synthesis of secondary metabolites, carbohydrate metabolism, energy metabolism, lipid metabolism, amino acid metabolism, metabolism of cofactors and vitamins, xenobiotic metabolism. Of note, multiple major pathways and processes were differentially expressed in the tumors from the three racial groups (Fig. 7 and Supplementary Figs. 4–6). Two-way ANOVA showed that most of the pathways analyzed including cellular processes, environmental and genetic information processing, carbohydrate and lipid metabolism and xenobiotic degradation were significantly elevated in tumors from Black women compared to breast tumors from both Asian and white women. However, no significant differences were observed in the tumors from Asian women as compared to the tumors from either Black or white women (Supplementary Figs. 4–6). Among the environmental information processing pathways, it is interesting to note that several oncogenic pathways were significantly upregulated in the tumors from Black women, including phosphatidylinositol signaling, mTOR signaling, calcium signaling and phosphotransferase system. ABC transporters were also upregulated in tumors from Black women, which are known to be responsible for the development of chemotherapy resistance in breast cancers. Surprisingly, VEGF signaling was downregulated in the same set. Two important oncogenic pathways, Notch and WNT, were also found to be upregulated in tumors from Black women (though not statistically significant) (Supplementary Fig. 5). Our group has shown important contributions of these pathways in the context of breast cancer-associated microbiome as well as racial disparities^22,30^. Collectively, we discovered some very interesting patterns and distinctive features in the microbial community that resides in breast tumors from Asian, Black and white women using a comprehensive metagenomic analysis of The Cancer Genome Atlas (TCGA) data.

### Integrated differential expression and pathway analysis of the breast tumors from Asian, Black, and White women exhibit unique racially associated genomic features

**Fig. 8:**
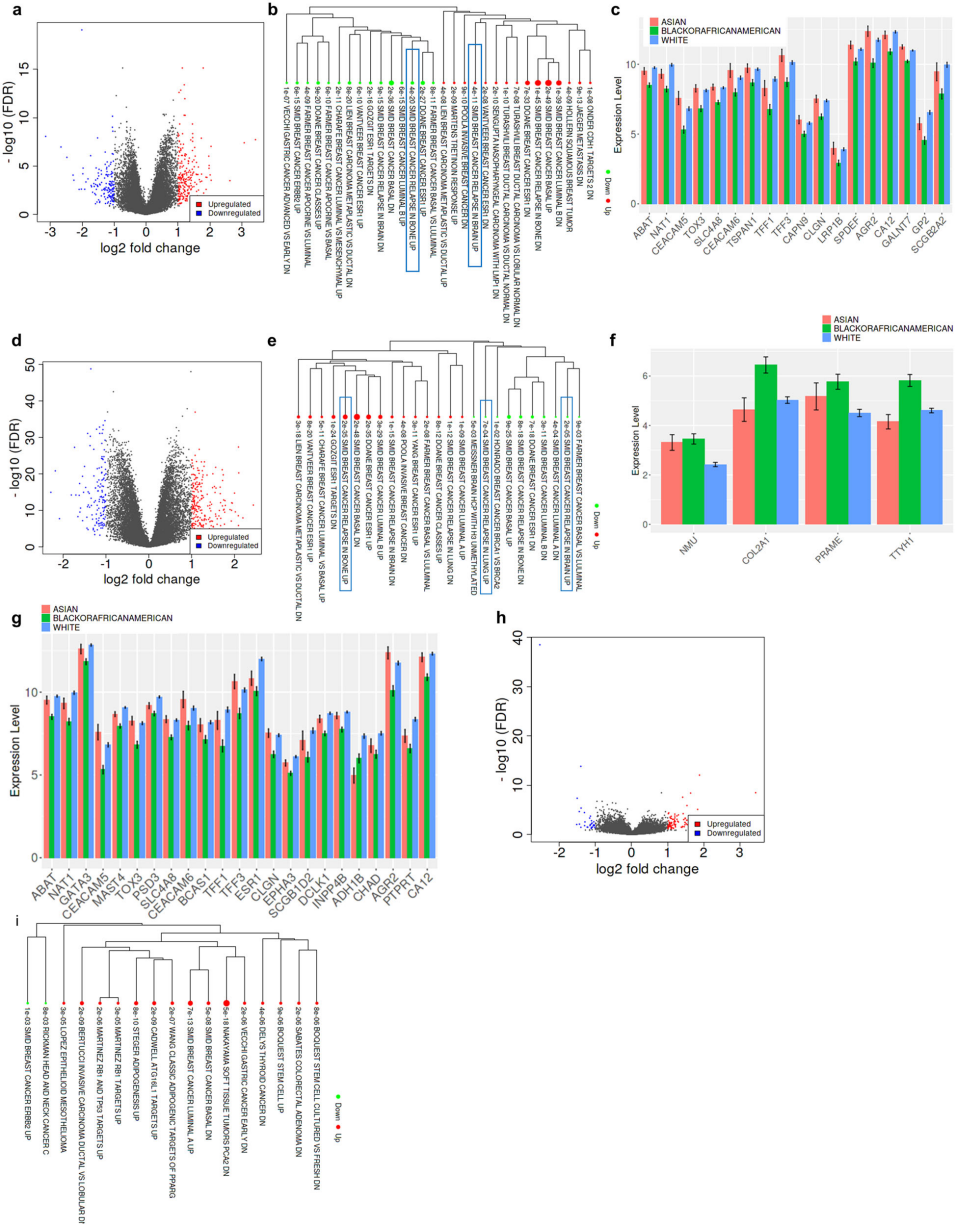
Breast tumors from different races exhibit unique racially associated genomic features. The gene expression profile of the 1018 primary breast tumors from Asian, Black and white women was examined using iDEP.94. **a** Volcano plot representing the differential gene expression of genes vs. false-discovery rate (FDR). **b** MA plot representing average gene expression vs. fold-change between Black and Asian groups. Differentially expressed genes were analyzed for enriched pathways between Black and Asian groups using Curated MSigDB dataset. Interactive network to visualize the relatedness of different pathways. **c** Bar graph represents the gene expression of selected genes involved in breast cancer bone metastasis from SMID-Breast cancer relapse in bone UP signature in different races. White vs. Black groups: **d** Volcano plot representing the differential gene expression of genes vs. false-discovery rate (FDR). **e** MA plot representing average gene expression vs. fold-change between white and Black groups. Differentially expressed genes were analyzed for enriched pathways between white and Black groups using Curated MSigDB dataset. Interactive network to visualize the relatedness of different pathways. **f** Bar graph represents the gene expression of selected genes involved in breast cancer lung metastasis in different races from SMID-Breast cancer relapse in Lung UP. Error bars represent standard deviation (SD). **g** Bar graph represents the gene expression of selected genes involved in breast cancer lung metastasis from SMID-Breast cancer relapse in Bone UP. White vs. Asian group. Error bars represent standard deviation (SD). **h** Volcano plot representing the differential gene expression of genes vs. false-discovery rate (FDR). **i** MA plot representing average gene expression vs. fold-change between white and Asian groups. Differentially expressed genes were analyzed for enriched pathways between white and Asian groups using Curated MSigDB dataset. Interactive network to visualize the relatedness of different pathways between white and Asian groups. For (**b**, **e**, **i**), Red dots represent upregulated pathways while green dots represent downregulated pathways. The size of the dots represents the number of the genes imparting pathway enrichment. The connection between the two pathways is represented by a line.

Since the pathological features of the tumor that directly impact tumor progression, reflect the molecular features, we analyzed the gene expression profile of the same (the ones queried for the microbial features) 1018 primary breast tumors from Asian (*n* = 65), Black (*n* = 257), and white (*n* = 696) women acquired from TCGA breast cancer dataset using iDEP.94. A total of 394 Differentially Expressed Genes (DEGs) were identified between breast tumors from Asian women vs. Black women (Fig. 8a) while 381 and 127 DEGs were identified to be significantly different between Black women vs. white women (Fig. 8d) and Asian women vs. white women (Fig. 8h), respectively. While multiple transcription factors and oncogenic drivers were identified that could potentially be the determinants of racial disparities in breast cancer outcomes, Enrichment tree analysis of differentially expressed genes using Curated MSigDB dataset indicated an upregulation of “SMID Breast Cancer Relapse in Bone UP” signature in breast tumors from Black women in comparison to tumors from Asian and white women in direct comparisons (Black vs. Asian, white vs. Black groups). We found that 6 and 11 genes contribute to breast cancer brain relapse in white vs. Black and Black vs. Asian groups comparison with an adjusted *p*-value of 1.63E-05 and 3.56E-11, respectively (Fig. 8b, e) (one-way ANOVA). Least bone metastasis was observed in Black women with breast cancer in comparison to both Asian and white counterparts, respectively (Fig. 8a–e). At the same time, higher expression of 4 important genes (NMU, COL2A1, PRAME, and TTYH1) that contribute to higher breast cancer lung metastasis was observed in the tumors from Black women compared to the tumors from white women with an adjusted *p*-value of 0.000664734 (Fig. 8f) (one-way ANOVA). Gene expression analysis of the contributory genes for lung metastasis indicated the highest expression in tumors from Black women compared to both white and Asian groups (Fig. 8c, g). This comprehensive analysis of breast tumors from Asian, Black, and white women uncovered that tumors in Black women have distinctive molecular features that may lead to the aggressive growth and metastatic progression.

To visualize the association between the genetic predictors of organotrophic metastasis between races, we did a spearman’s rank correlation analysis between expression levels of differentially expressed genes and microbial biomarkers revealing some interesting patterns (Table 1 and Supplementary Figs. 7–8). In breast tumors from both Asian and Black groups, TGFB3 showed a significant negative correlation with most of the microbial biomarkers including Xanthomonas, Amycolatopsis, Pseudomonas and Succinomonas. While some microbes showed positive correlation, others showed negative correlations with VEGF A, B, and C across all races. GLI1 showed a significant positive correlation with Terrabacter in tumors from Asian and Black women while it was found to be negatively correlated with Succinomonas in both the races. These results indicate a possible role of intratumor microbiota in tumor vasculogenesis and thus warrant further investigation.

**Table 1:** Spearman coefficients indicating correlation between gene expression and abundance of microbial biomarkers.

Black			
Bacteria	Gene	*r* ^2^	*p*-value
Xanthomonas	TOX3	−0.18908	0.015964
	CLGN	−0.20546	0.008718
	TGFB3	−0.15429	0.048542
Amycolatopsis	NAT1	−0.17375	0.024731
	TSPAN1	−0.15615	0.043886
	CAPN9	−0.17017	0.029373
	CLGN	−0.18576	0.016568
	SPDEF	−0.2	0.009342
	GALNT7	−0.18202	0.018919
	GP2	−0.13905	0.056805
	NOTCH1	0.176936	0.02177
	GLI1	−0.19203	0.013477
	TGFB3	−0.17982	0.01968
Aminiphilus	VEGFA	−0.17279	0.029402
	VEGFC	0.163155	0.038644
	GLI1	0.222058	0.004771
Clostridioides	ABAT	0.0779	0.035727
	TGFB2	0.082333	0.026426
Pseudomonas	SLC4A8	−0.19737	0.010569
	TSPAN1	−0.16628	0.031227
	CAPN9	−0.17616	0.023193
	CLGN	−0.15037	0.049627
	SPDEF	−0.20384	0.007671
	AGR2	−0.17206	0.026648
	CA12	−0.16597	0.030046
	GALNT7	−0.15349	0.046335
	GLI1	−0.15861	0.039432
	TGFB3	−0.21952	0.004135
Terrabacter	VEGFA	−0.16108	0.036425
	VEGFC	0.186959	0.014638
	GLI1	0.239231	0.001733
	IL6	0.187867	0.014744
Clostridioides	TOX3	0.160857	0.040243
	VEGFB	−0.16784	0.032231
	TGFB1	−0.24813	0.001405
Succinimonas	SPDEF	−0.16398	0.034767
	VEGFB	−0.16363	0.035162
	GLI1	−0.18257	0.018918
	TGFB1	−0.17821	0.021212
Catellicoccus	ABAT	0.1943	0.012944
Leucobacter	TOX3	0.162761	0.03851
	CA12	0.216804	0.005439
	TGFB1	−0.22384	0.004192
Rhizobium	CA12	0.249773	0.001302
	SCGB2A2	0.155113	0.048732
	TNF	−0.1886	0.018006
Rhodococcus	TFF1	0.290414	0.000214
	VEGFA	−0.18512	0.019104
	VEGFB	0.153734	0.051527

White			
Bacteria	Gene	*r* ^2^	*p*-value
Leucobacter	GLI1	0.093924	0.011864
	ABAT	−0.07797	0.036342
	SPDEF	−0.07314	0.049327
Rhodococcus	TGFB3	0.079361	0.033367
Catellicoccus	CEACAM6	0.074453	0.046731
Clostridioides	ABAT	0.0779	0.035727
	TGFB2	0.082333	0.026426
Pseudomonas	SLC4A8	0.076908	0.038024

Asian			
Bacteria	Gene	*r* ^2^	*p-*value
Aminiphilus	NAT1	−0.27947	0.043789
Pseudomonas	VEGFB	0.282372	0.042541
	VEGFC	0.270225	0.052689
	TGFA	−0.26572	0.059485
	TGFB2	0.279784	0.046764
Terrabacter	GLI1	0.288815	0.028795
Clostridioides	NOTCH1	−0.27468	0.035259
Succinimonas	SLC4A8	−0.26501	0.042512
	SPDEF	−0.32803	0.010508
	TGFB1	−0.33851	0.008157
	TGFB3	−0.26513	0.038925
	IL6	−0.35697	0.005514
	NOTCH1	−0.22884	0.078631
	GLI1	−0.24864	0.053328
Catellicoccus	SPDEF	−0.46604	0.000199
	AGR2	−0.25476	0.051507
Leucobacter	IL6	−0.30843	0.026107
Rhizobium	VEGFC	−0.31929	0.017498
	TGFB1	−0.33295	0.012999
Rhodococcus	SLC4A8	−0.46198	0.000643
	AGR2	−0.2764	0.045127
	TGFB2	0.388104	0.004469

## Discussion

Despite remarkable therapeutic advancements in breast cancer, racial disparities in clinical outcomes persist. Some molecular factors such as alterations in oncogenic signaling or tumor suppressor pathways have been implicated in disparate tumor progression in some racial groups^10,15,22,46,47^. We recently discovered that triple-negative breast cancer (TNBC) cells from Black women display higher growth and migratory behavior with elevated stemness potential than those from white women. We also observed an aberrant activation and functional interaction of Gli1 and Notch1 in TNBC tumors from Black women compared to TNBC tumors from white women^22^. It is now appreciated that tumor progression is not only guided by the tumor-centric alterations but supracellular patterns encompassing various tumor microenvironment features may also play an important role. Heterogeneity of the tumor microenvironment is further complicated with the presence of microbiota residing in tumor and immune cells. In this study, we aimed to put forth a comprehensive and simultaneous analysis of gene expression and metagenomic profiles in a large population of breast cancer patients comprising multiple races. We noted a significantly higher accumulation of activated dendritic cells (aDC), B cells, epithelial cells, megakaryocyte–erythroid progenitors (MEP), mesenchymal stem cells (MSCs), Sebocytes, and Th1 cells in breast tumors from Black women compared to tumors from white women. Breast tumors in Asian as well as Black women have been shown to be more immune-active compared to white women, however, in Black women, the TME is thought to be more pro-tumorigenic with increased microvasculature and more involvement of M2-macrophages and T regulatory cells^2,48^. Changes in immune microenvironment are closely intertwined with alterations in cytokines. While many cytokines including IL1β, IL6, IL2, and IL12 did not show any significant race-specific differences, IFNγ exhibited race-specific accumulation. Among the inflammatory chemokines CXCL9, CXCL10, and CXCL11 primarily stimulated by IFNγ, CXCL9 was found to be significantly overexpressed in tumors from Black women compared to others. Evaluation of the cytokine profiles of Black and white women with breast cancer uncovered a higher expression of Resistin and IL6 in the serum samples of Black women. Resistin could promote higher growth, migration and invasion of MDA-MB-468 (a TNBC cell line from a Black woman) compared to MDA-MB-231 (a TNBC cell line from a white woman) cells^20^. Race-associated disparities were also observed in the polymorphisms of IL10 and IFNγ in African and mixed-race population groups of South Africa with IFNγ expression correlating with the race across various population groups^21^. Several important differences in resident immune cells and cytokines were noted in breast tumors from different races that can potentially alter tumor initiation and progression.

Another interesting observation of our study is the race-specific differential accumulation of microbes that are known to be over-represented in breast tumors compared to normal breast tissue. Acinetobacter, Citrobacter, Enterobacter, Staphylococcus, Paracoccus, and Akkermansia were differentially abundant in breast tumors from Black women compared to white group whereas Actinomyces and Veillonella were plentiful in breast tumors from white women in comparison to Black women. Breast tumors from Asian women exhibited very few alterations. In addition to microbes with known functions, our study uncovered various race-associated microbial biomarkers whose direct role in human health and disease is yet to be uncovered. Pseudomonas and Methylobacter, among others, were recognized as the biomarkers in breast tumors from Asian women. P*seudomonas aeruginosa*–mannose-sensitive hemagglutinin (PA-MSHA) has been shown to have an antiproliferative effect on breast cancer and was investigated in a clinical trial for the treatment of breast cancers^49^. Both Pseudomonas and Methylobacter were found to be enriched in breast tumors^24^. Genus Amycolatopsis, identified as a biomarker for breast tumors from Black women, is known to produce a wide range of antibiotics including vancomycin, which is effective against antibiotic resistant infections^50^. Anaerovorax, another biomarker identified for tumors from Black women, is known to metabolize putrescine to acetate, butyrate, molecular hydrogen and ammonia^51^. Of note, putrescine is one of the important polyamines that has been shown to drive breast cancer development^52–54^.

The role of microbiota in breast cancer is only starting to be revealed and the racial differences have hardly been considered. The microbiome is cumulatively shaped by various environmental, lifestyle, dietary, disease and treatment-associated factors^37–39^. It is important to note that Asian, Black and white women face different socioenvironmental stressors (e.g., poverty, racism, discrimination, etc.) in the United States. It is plausible that these differences in exposures may have consequences in differences in the tumor microenvironment. Microbiota is an important regulator of immunity, hormone metabolism and energetics and a dysbiosis significantly impacts cancer risk, shapes the tumor microenvironment and determines therapy response. Two recent studies examined the race-specific microbiota of breast cancers^40,41^. Slightly constrained by the sample size and depth of sequencing, they lack species and strain level information but reveal some very interesting patterns. Starlard-Davenport and group characterized the microbiota of 64 breast tumor samples and compared it to 11 adjacent normal samples^40^. Classifying by the race, family Xanthomonadaceae was the most abundant member in breast tumors from Non-Hispanic white (NHW) women, whereas genus Ralstonia was most abundant in breast tumors from Non-Hispanic Black (NHB) women. Tumors from NHW women were richer in Phylum Bacteroidetes compared to NHB women^40^. Racial differences in the breast microbiota were also compared by Vishwanatha and colleagues using breast tumors from 23 Non-Hispanic white women and 10 Non-Hispanic Black women and their normal adjacent breast tissue^41^. In TNBC tumors from Black women, both Shannon diversity and evenness were reduced in tumor tissue compared to adjacent normal tissue while the trend was reversed in TNBC tumors from white women. Phylum Bacteroidetes was significantly over-represented in TNBC from white women compared to adjacent normal tissue (*p* = 0.03) while in TNBC from Black women, phylum Actinobacteria (*p* = 0.03) and unclassified genus of Bradyrhizobiaceae (*p* = 0.03) were underrepresented. Another interesting observation was the significant underrepresentation of phylum Thermi in TNBC from Black women compared to their adjacent normal tissue and relative to the white group where no significant difference between tumor and adjacent normal tissue was observed^41^. Notably, the two differentially abundant members of phylum Thermi and genera Ralstonia are known to be highly resistant microbes. While Ralstonia is known to be resistant to most antibiotics including carbapenems^55^, representatives of phylum Thermi or Deinococcus-Thermus are extremophiles, highly resistant to ionizing and non-ionizing radiation as well as oxidative stress. These organisms have also been suggested to be potentially important in designing cancer therapies^56^. In our analysis, absolute read count of phylum Thermi was found to be significantly higher in tumors from Black women compared to both Asian and white group. Utilizing a larger set of samples (*n* = 1018) encompassing tumors from Asian, Black, and white women, our results provide an insight into the differential abundance of specific microbes in different races.

Our gene expression analysis showed a significant difference in metastasis predictors between races. PICRUSt analysis showed a differential enrichment of multiple pathways and processes including important oncogenic and xenobiotic metabolism pathways among different races. These could have implications in drug activation and detoxification. Our study is limited by the fact that the data obtained is from a retrospective analysis of whole genome and we do not have strain level information. As many microbes like *E. coli* and *B. fragilis* are known to have multiple strains, some of which are pathogenic while others are normal commensals, our study may not have identified all the important strains. We decided to focus on the alpha and beta-diversity measures and uncovered important differences among various groups. In addition, we identified important microbial biomarkers at the order, family and genus level. Future studies with more in-depth sequencing will be able to identify key microbial strains. Another caveat is the noninclusion of ethnicity information and ancestry markers owing to the retrospective nature of this analyses as they can capture some important social, cultural, geographical and economic issues related to health disparities. The present study highlighted the differences in various sets of microbiota, genomic and metabolic features among different self-reported race groups. In future, studies including the ethnicity information and ancestry markers would be able to further delineate these features. Nonetheless, our study paves the path for further explorations as future studies examining both ancestry and race may help guide their relationship to the tumor microenvironment. We have made a broad classification here between races irrespective of subtype specificity, menopausal status and metastatic status of the patients, which will be considered in forthcoming studies. Our study explicitly presents that breast tumors from women of different races possess specific cellular patterns in the tumor microenvironment, harbor distinct sets of microbiota, and have unique genomic features. We uncover the differential abundance of microbes with known biological importance as well as many microbes whose importance in breast cancer is currently unknown. Several epidemiological studies have shown the race-specific differences in breast cancer initiation, progression and outcomes, but the complexities of underlying biology are still unclear. Our study is an important step towards examining factors beyond the usual tumor-centric approaches, as it paves the way for including race-related microbial dysbiosis in clinical decisions.

## Methods

### Dataset, data acquisition, and quality control

We examined the genomic and metagenomic data from The Cancer Genome Atlas (TCGA) cohort encompassing 1018 breast cancer patients from different races. This data is publicly available. According to the National Human Genome Research Institute, race is a social construct to group people. Race divides human populations into groups often based on physical appearance, social factors and cultural backgrounds (NHGRI, NIH). TCGA data includes self-reported race groups in the US. Self-reported race in the US is largely associated with socioeconomic status, geographic location and access to health care among other factors; and has been used by multiple studies to determine the biological differences among race groups^22,57,58^. Our analyses include self-reported race groups-Asian, Black and white. In the TCGA Asian dataset, 74% of samples were collected in Vietnam, 9% in the US, 3% in Pakistan while remaining 15% came from repositories around the US. In the TCGA Black dataset, only 2 out of 257 Black breast cancer patients were Hispanic, which accounts for less than 0.8% of the sample population. Hence, we consider this dataset to be a fair representation of the non-Hispanic Black group.

Normalized RNA sequencing TCGA breast cancer dataset was retrieved from Broad GDAC Firehose (http://firebrowse.org/?cohort=BRCA&download_dialog=true%27). For metagenomic information, Kraken-TCGA-Raw-Data (*n* = 17,625) and Metadata-TCGA-Kraken-17625-Samples were downloaded from the repository (ftp://ftp.microbio.me/pub/cancer_microbiome_analysis/)^59^. Breast cancer samples were filtered out and categorized by race (Asian, Black, and White). Samples without race information were excluded. The raw data was then curated to obtain bacterial OTU table for samples with Asian, Black and white group using Microsoft Excel. For this study, we only used the data from primary tumors (Supplementary Data 1). For tumor heterogeneity analysis, pre-calculated enrichment scores for all cell types for the entire TCGA datasets for all cancers was downloaded from the website https://xcell.ucsf.edu/. Data was curated to extract breast cancer data and was clustered into three racial groups, Asian, Black and white. Samples without race information were eliminated.

### Gene expression analysis

The preprocessed dataset was then analyzed using integrated differential expression and pathway analysis (iDEP.94) pipeline. iDEP.94 is an online web-based interface designed for RNA sequencing or microarray data analysis. Normalized gene expression data was uploaded as an input file for human species. Gene expression changes were evaluated between 3 groups as white vs. Black, white vs. Asian and Black vs. Asian, respectively. Volcano plots and MA plots were used for evaluating the data distribution between different races. Race-wise comparison of differentially expressed genes was performed for Enrichment tree and network analysis using Curated MSigDB dataset.

### Metagenomic analysis

Microbial community composition between breast tumors from Asian, Black and white women were compared by measuring Alpha (Dominance, Berger-Parker richness, Heip evenness, Simpson evenness, Fisher alpha index, Shannon entropy, Chao1 index and abundance coverage-based estimator (ACE)) using QIIME2–2021.11. Principle Coordinate Analysis (PCoA) based on Bray–Curtis, Jensen-Shannon, Jaccard and correlation matrix were performed to determine Beta diversity (Supplementary Data 2). To identify significantly different bacteria as known as microbial biomarkers among races, Linear discriminant analysis Effect Size (LEfSe) was performed at Order, Family and Genus levels using Huttenhower Lab Galaxy Server^60^. Distinguishing members were identified based on the threshold Linear Discriminant Analysis (LDA) score for discriminative feature 2 at alpha value for Kruskal–Wallis test 0.05, and alpha value for pairwise Wilcoxon test between subclasses 0.05 using all against all strategy for analysis. To understand the probable metabolic contributions of the tumor-specific microbial communities, Phylogenetic Investigation of Communities by Reconstruction of Unobserved States (PICRUSt) was performed using QIIME2–2021.11 followed by Kruskal–Wallis test and Dunn’s post-test between all pairs to compare between races. All heatmaps and PCoA graphs were constructed using R version 4.1.2. Other statistical analyses were performed and graphs drawn using GraphPad Prism.

### Reporting summary

Further information on research design is available in the Nature Research Reporting Summary linked to this article.

## Supplementary information

Supplementary information

Reporting Summary

Supplementary Data 1

Supplementary Data 2

## Data Availability

Normalized RNA sequencing TCGA breast cancer dataset is available on Broad GDAC Firehose (http://firebrowse.org/?cohort=BRCA&download_dialog=true%27). Kraken-TCGA-Raw-Data (*n* = 17,625) and Metadata-TCGA-Kraken-17625-Samples are available on the repository (ftp://ftp.microbio.me/pub/cancer_microbiome_analysis.

## Code availability

The custom code used in this study are available in Supplementary Data 1. Readers are encouraged to directly contact the corresponding author for additional information.
