# Author Correction: Concomitant analyses of intratumoral microbiota and genomic features reveal distinct racial differences in breast cancer

**DOI:** 10.1038/s41523-023-00521-6

**Published:** 2023-03-23

**Authors:** Sheetal Parida, Sumit Siddharth, Yuqing Xia, Dipali Sharma

**Affiliations:** Department of Oncology, Johns Hopkins University School of Medicine and Sidney Kimmel Comprehensive Cancer Center at Johns Hopkins, Baltimore, MD USA

Correction to: *npj Breast Cancer* 10.1038/s41523-023-00505-6, published online 26 January 2023

In this article the wrong figure appeared as Fig. 3; the figure should have appeared as shown below. Additionally, the Code Availability as well as Supplementary Data 1 and Supplementary Data 2 were missing from this article and have now been uploaded. The original article has been corrected.
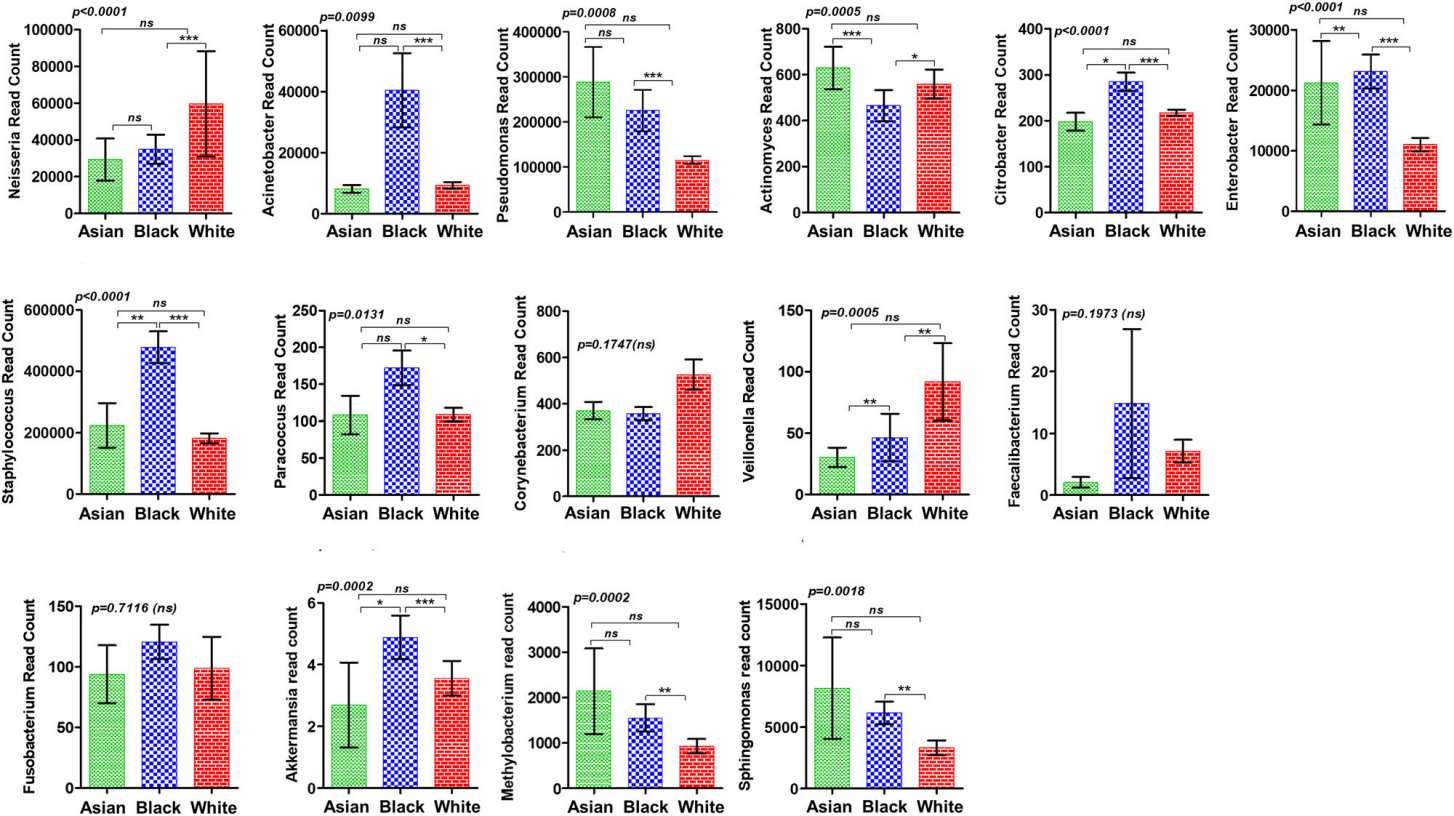


## Supplementary information

Supplementary Data 1

Supplementary Data 2

## Data Availability

The custom code used in this study are available in Supplementary Data 1. Readers are encouraged to directly contact the corresponding author for additional information.

