# Supplementary material for: Concomitant analyses of intratumoral microbiota and genomic features reveal distinct racial differences in breast cancer
Source: NPJ Breast Cancer. 2023 Jan 26;9:4. doi: 10.1038/s41523-023-00505-6 (PMC9880005; doi:10.1038/s41523-023-00505-6)
Supplement: Supplementary file 1 — Supplementary information [file 41523_2023_505_MOESM1_ESM.pdf]

Supplementary Information

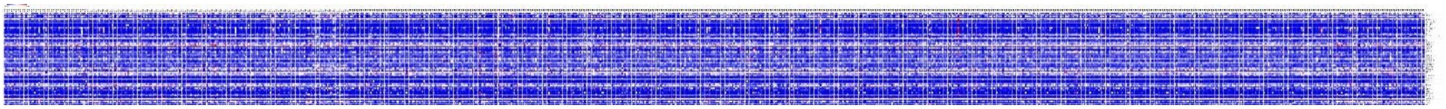

Supplementary Figure 1 Comparative landscape of breast tumors among different races. The TCGA data for all breast cancers was analyzed for their 64-cell type signature using web-based tool xCell. Pre-calculated enrichment scores for all cell types for entire TCGA dataset for all cancers was downloaded from the website https://xcell.ucsf.edu/. Data was curated to extract breast cancer data and was clustered into three races; Asian, Black and white. Heatmap presents the comparative cellular landscape of breast tumors from Asian, Black and white women.

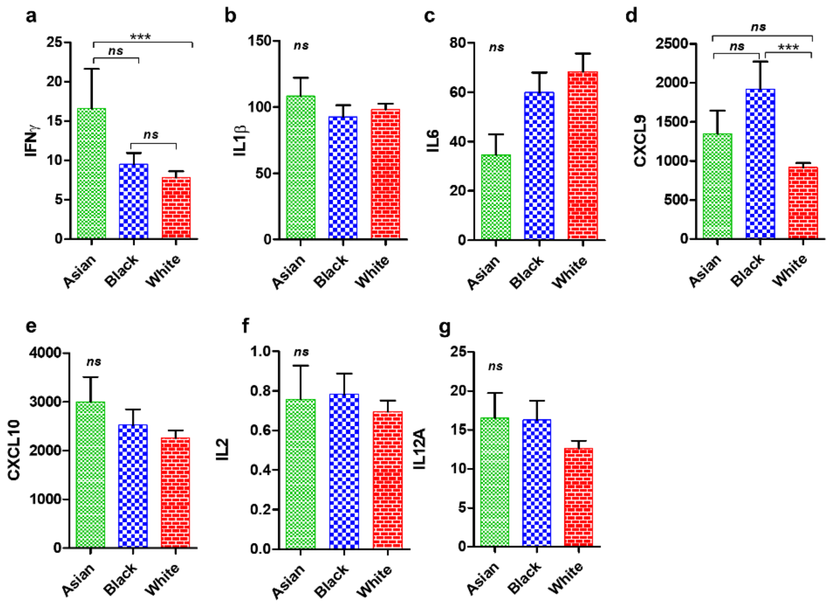

Supplementary Figure 2 Gene expression levels of Th1 induced cytokines in breast tumors across races. (a-g) Bar graphs present the levels of IFNγ, IL1β, IL6, CXCL9, CXCL10, IL2 and IL12A in the tumors from Asian, Black and white women. Significance calculated by one-way ANOVA followed by Bonferroni post-test. ***p<0.0001, **p<0.001, *p<0.05. Error bars represent standard deviation (SD).

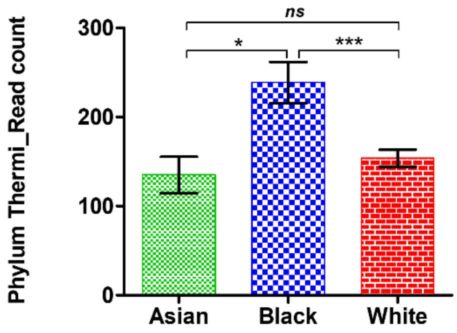

Supplementary Figure 3 Phylum Thermi shows differential enrichment across races. The bar graph shows absolute read count of phylum Thermi in breast tumors from Black women compared to both Asian and white women. Significance calculated by one-way ANOVA followed by Bonferroni post-test. ***$p<0.0001$, *$p<0.05$. Error bars represent standard deviation (SD).

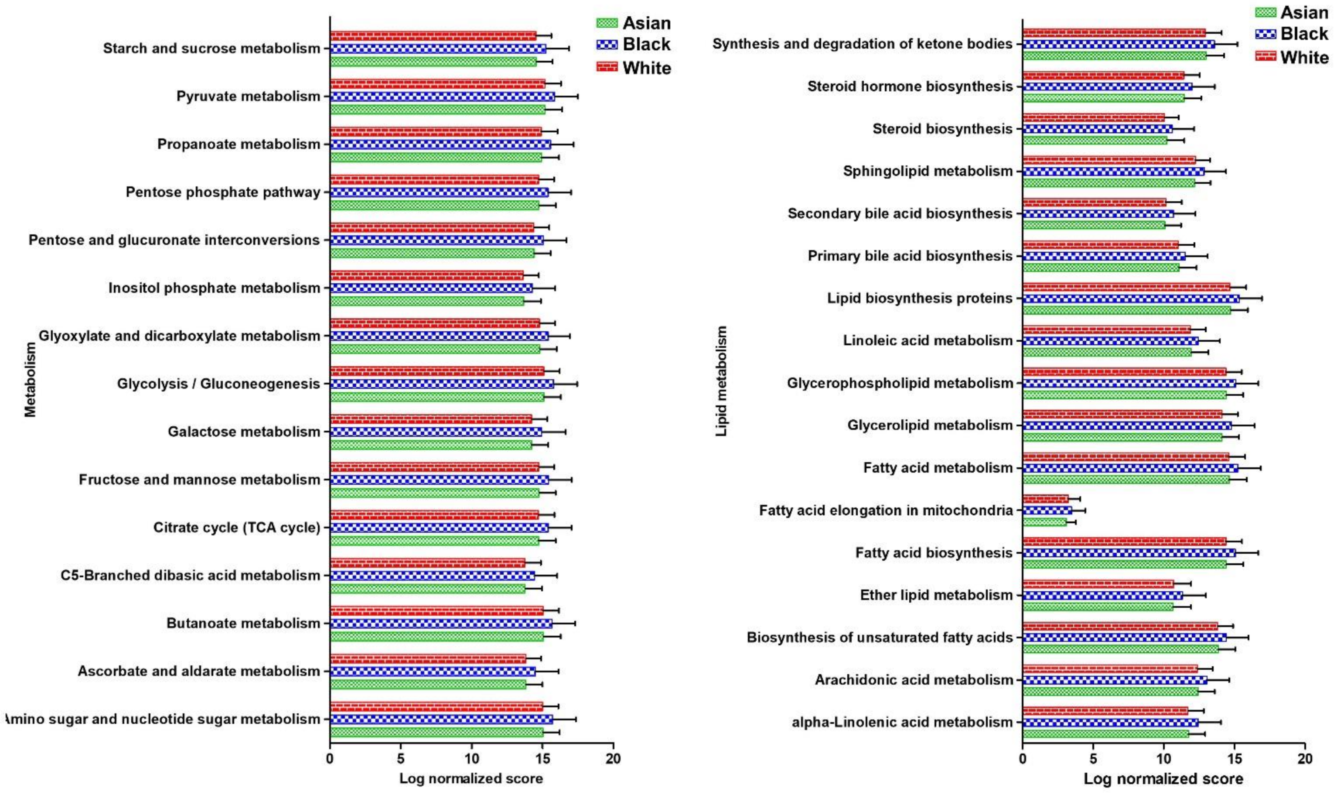

Supplementary Figure 4 Selected KEGG pathways are enriched in breast tumors from different races. PICRUSt analysis showing an enrichment of selected metabolic pathways. Statistical significance denoted by *p<0.05, **p<0.001, ***p<0.0001. Comparisons shown between Asian vs Black and Black vs white group. All comparisons were non-significant for Asian vs white. Significance calculated by two-way ANOVA followed by Bonferroni post-test. Error bars represent standard deviation (SD).

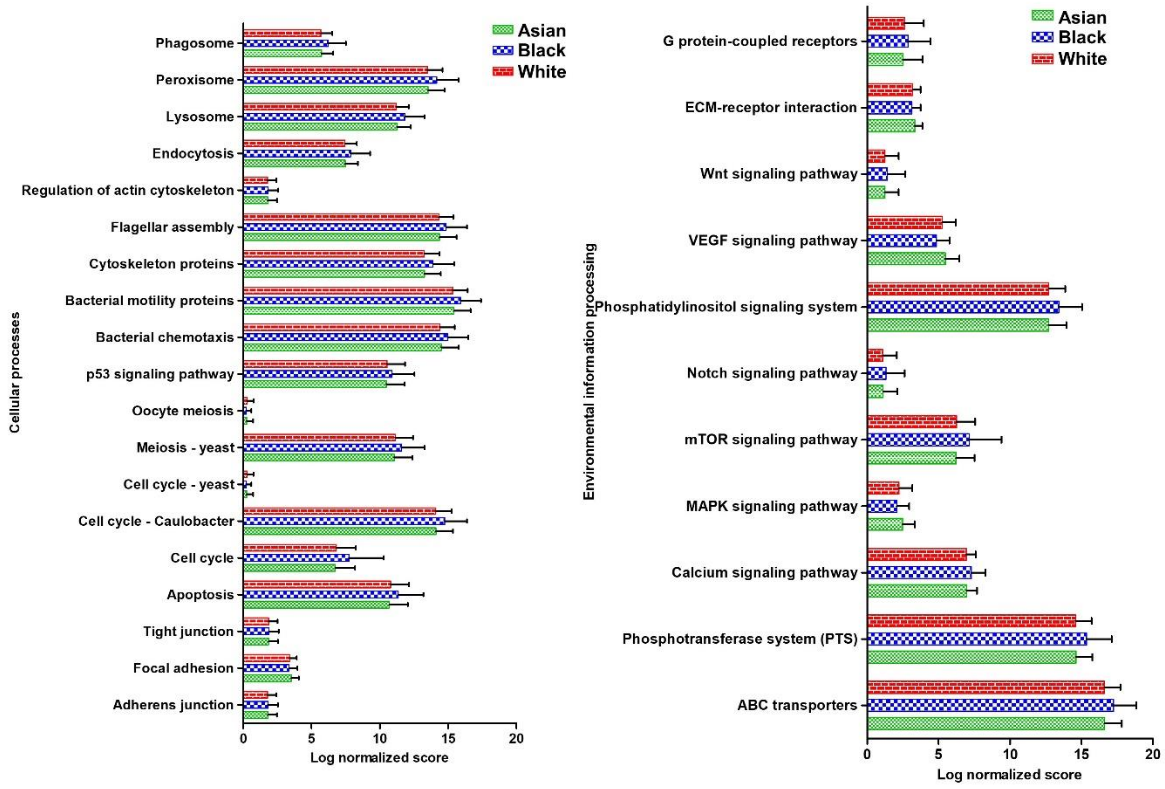

Supplementary Figure 5 Selected KEGG pathways are enriched in breast tumors from different races. PICRUSt analysis showing an enrichment of selected cellular and environmental information processing pathways. Statistical significance denoted by *p<0.05, **p<0.001, ***p<0.0001. Comparisons shown between Asian vs Black group and Black vs white group. All comparisons were non-significant for Asian vs white group. Significance calculated by two-way ANOVA followed by Bonferroni post-test. Error bars represent standard deviation (SD).

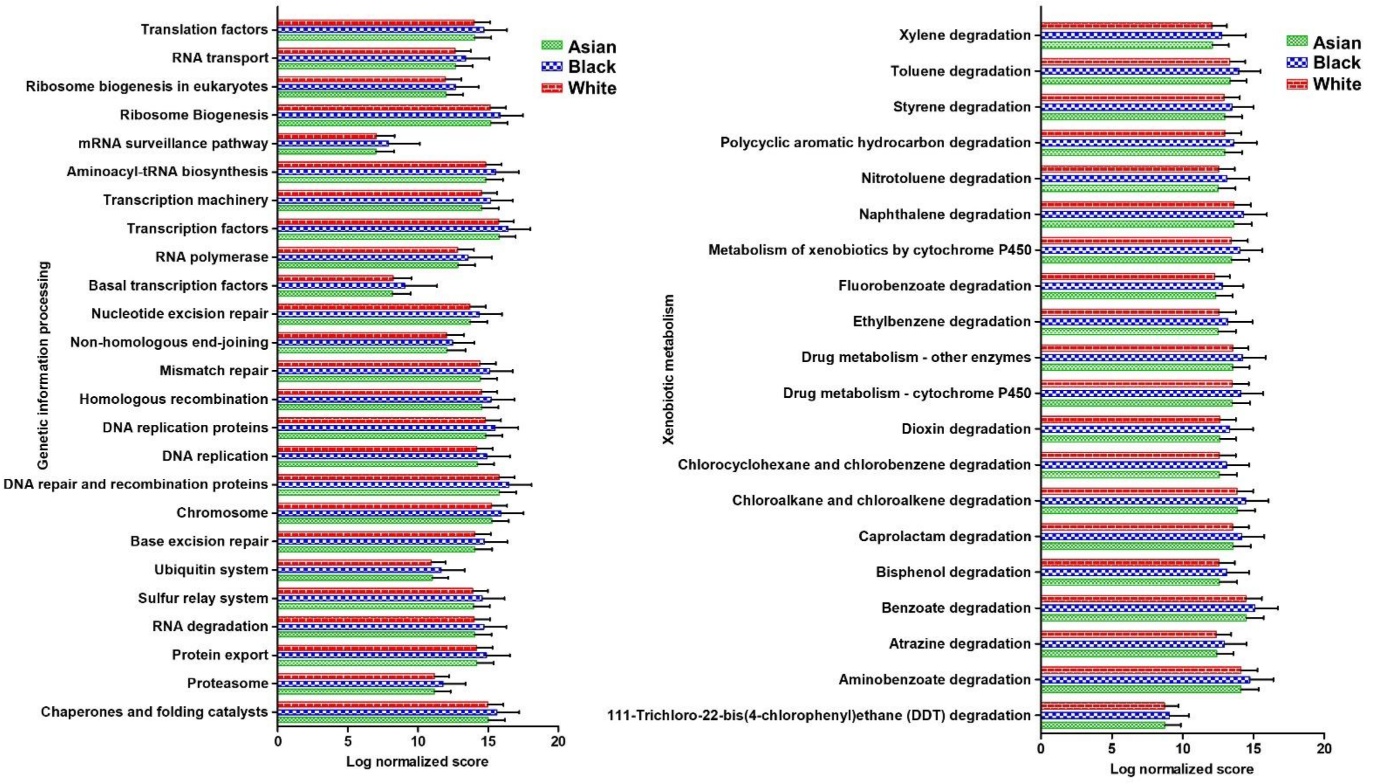

Supplementary Figure 6 Selected KEGG pathways are enriched in breast tumors from different races. PICRUSt analysis showing an enrichment of selected genetic information processing and xenobiotic metabolism pathways. Statistical significance denoted by *p<0.05, **p<0.001, ***p<0.0001. Comparisons shown between Asian vs Black and Black vs white groups. All comparisons were non-significant for Asian vs white groups. Significance calculated by two-way ANOVA followed by Bonferroni post-test. Error bars represent standard deviation (SD).

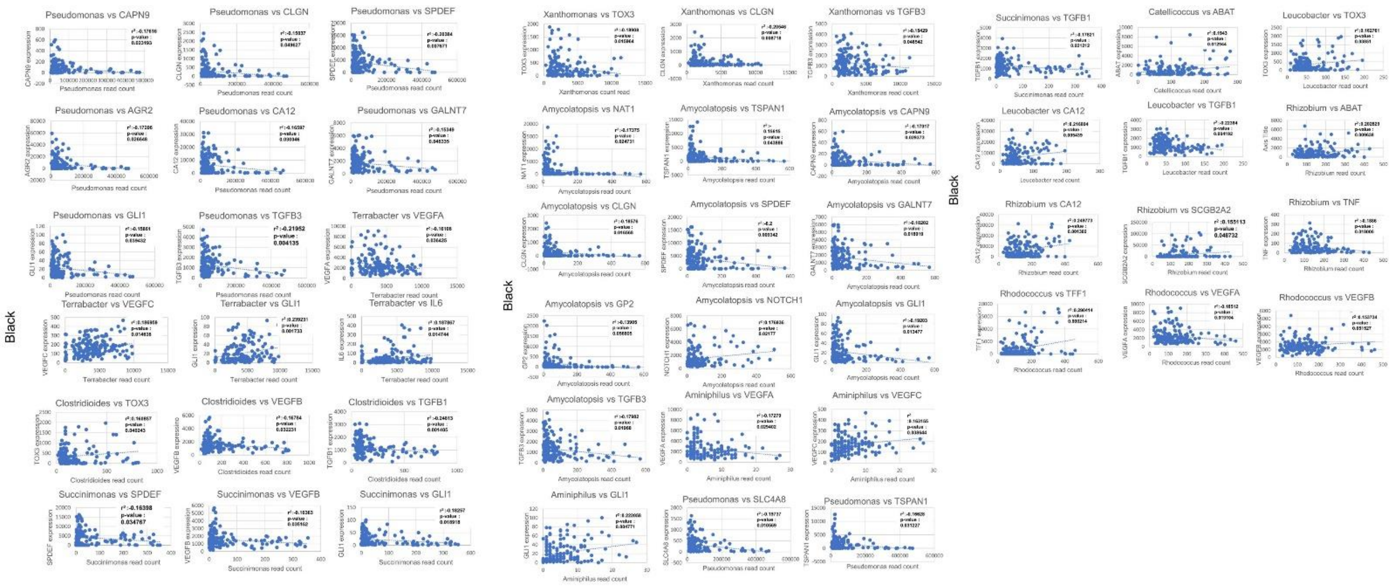

Supplementary Figure 7 Correlation analysis between expression levels of differentially expressed genes and microbial biomarkers. Spearman coefficients indicating correlation between gene expression and abundance of microbial biomarkers in breast tumors from Black women.

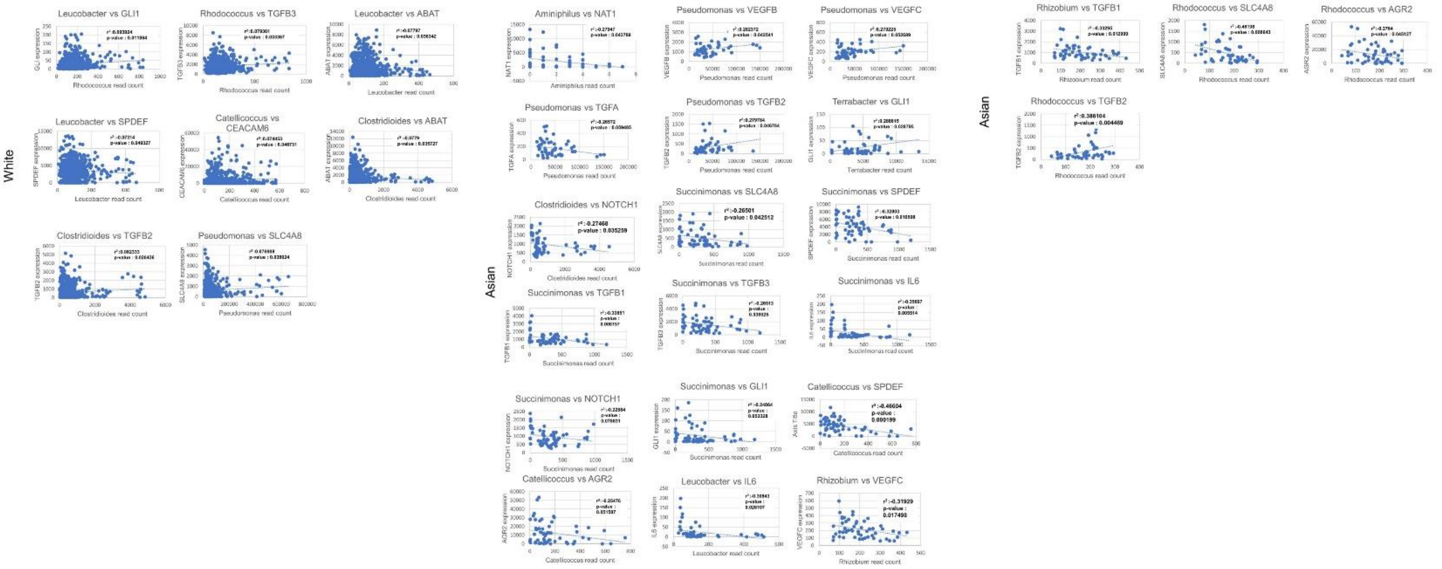

Supplementary Figure 8 Correlation analysis between expression levels of differentially expressed genes and microbial biomarkers. Spearman coefficients indicating correlation between gene expression and abundance of microbial biomarkers in breast tumors from white and Asian women.
